# Unintentional ingestion of a high dose of acenocoumarol in a young child

**DOI:** 10.1136/bcr-2020-240365

**Published:** 2021-04-01

**Authors:** Martijn R Brands, Jelmer Sytema, Marinus van Hulst, Arvid WA Kamps

**Affiliations:** 1 Paediatrics, Martini Hospital, Groningen, The Netherlands; 2 Clinical Pharmacy and Toxicology, Martini Hospital, Groningen, The Netherlands; 3 Department of Health Sciences, University Medical Centre Groningen, Groningen, The Netherlands

**Keywords:** haematology (drugs and medicines), paediatrics (drugs and medicines), poisoning, haematology (incl blood transfusion), warfarin therapy

## Abstract

Acute intoxication with a vitamin K antagonist may cause serious coagulopathy. We report the accidental ingestion of a high dose of acenocoumarol in a young child. Two intravenous administrations of 5 mg of vitamin K, in combination with fast and repeated administration of activated charcoal and sodium sulfate, were sufficient to prevent coagulopathy and related symptoms, despite a confirmed elevated blood acenocoumarol concentration (260 µg/L).

## Background

Vitamin K antagonists (VKAs) are frequently prescribed for the treatment and prevention of venous and arterial thromboembolic events in adults. Internationally, warfarin is the most prescribed VKA. In several European countries, including the Netherlands, acenocoumarol and phenprocoumon are predominantly used instead.^1 2^

Because of the narrow therapeutic window, clinicians are regularly confronted with a supratherapeutic international normalised ratio (INR) in patients using VKA. However, an acute overdose of a VKA is less common and needs a different approach. Only a few case reports have been published on this topic, and almost all concern adults.^3–7^ Although some cases of accidental poisoning of children with anticoagulant rodenticides (superwarfarins) have been described, to our knowledge, no cases have been published of young children ingesting an overdose of a medicinal VKA.^8–12^

We report a case of a 20-month-old child who was successfully treated with intravenous vitamin K after the accidental ingestion of a high dose of acenocoumarol.

## Case presentation

A 20-month-old girl (13 kg) without any relevant medical history was brought to our emergency department 1 hour after the ingestion of a large number of tablets of the VKA acenocoumarol. The girl had been playing at her aunt’s house without adult supervision for a short time when she was found in a puddle of her vomit, in which at least 50 tablets of 1 mg acenocoumarol were identified. Although quite startled, she was conscious when discovered and was not vomiting anymore. Considering the original number of tablets in the medicine bottle, the ingested amount was estimated to be 60–90 mg, equivalent to approximately 110–170 mg warfarin in terms of clinical effect and potency.^13^

## Investigations

At presentation, all vital signs were normal. Baseline values of the coagulation parameters—the INR, the activated partial thromboplastin time and the prothrombin time—were determined. All values were within the normal range (table 1). Other laboratory values measured at baseline, including whole blood count, electrolytes and liver functions, were within the normal ranges.

**Table 1:** Laboratory results and interventions

	Reference value^22^	Hours after ingestion											
		1	2	4	7	8	13	19	21	28	40	52	64
INR	0.9–1.1*		1.1		1.1		1.1		1.0	1.1	1.0	1.0	1.0
aPTT (s)	24–29		29		31					33		34	
PT (s)	11–15		10.9		11.2					13.5		12.7	
Intervention		Activated charcoal		Vitamin K 5 mg		Activated charcoal		Vitamin K 5 mg					

*For children not using anticoagulant drugs.

aPTT, activated partial thromboplastin time; INR, international normalised ratio; PT, prothrombin time.

## Treatment

Immediately after arrival at the emergency department, activated charcoal (1 g/kg body weight) and sodium sulfate (0.5 g/kg body weight) were administered to prevent absorption and to promote laxation, respectively. Administration of activated charcoal and sodium sulfate were repeated once. We chose to proactively administer 5 mg of vitamin K twice, with an interval of 12 hours, to antagonise the effects of acenocoumarol. All interventions are presented on a timeline (table 1). To confirm the intake of the VKA, the blood level of acenocoumarol was measured in a blood sample which was taken 7 hours after the ingestion. The acenocoumarol level was 260 µg/L (liquid chromatography with tandem mass spectrometry). As a comparison, in adults, the therapeutic plasmatic concentration of acenocoumarol in steady state is in the range of 30–90 µg/L.^2^

## Outcome and follow-up

The child was admitted to the paediatric department. We chose to measure the INR every 6 hours on the first day and two times per day on the following 2 days. All coagulation parameters remained within the reference ranges. No complications occurred during the admission, and follow-up after 6 weeks remained uneventful.

## Discussion

Acute overdoses of acenocoumarol are relatively rare. We could not find any case reports or clinical studies regarding the treatment of accidental ingestion of high doses of VKA in (small) children.

To avoid haemorrhage and the potential need of treatment with prothrombin complex concentrate, we decided not to wait for the INR to rise. We proactively administered 5 mg of vitamin K to antagonise the effects of acenocoumarol. Vitamin K was given as a slow intravenous injection, since orally administrated vitamin K would bind to activated charcoal. We used the mixed micellar preparation of vitamin K, which has a lower risk of anaphylactoid hypersensitivity reactions than the older Cremophor EL-solubilized (PEO-CO) preparation.^14 15^

Our dosage of 5 mg vitamin K intravenously (0.38 mg/kg) was based on the Dutch guideline for coumarin intoxications, in which the administration of 1 to 10 mg (oral) vitamin K is recommended in children aged 1–14 years, not differentiated by age or body weight.^16^ The dose was comparable to a dose of 0.3 mg/kg vitamin K IV (maximum single dose 10 mg) as advised in cases of major bleeding in children with anticoagulant rodenticide poisoning.^17^

VKAs block the function of the enzyme vitamin K epoxide reductase (VKOR), which is needed to ‘recycle’ vitamin K epoxide—the biological inactive form of vitamin K—back to active vitamin K. Vitamin K serves as an essential cofactor for the activation of several proteins, including the coagulation factors II, VII, IX and X and protein C and S. Thus, through the selective inhibition of VKOR, administration of VKA results in a deficiency of vitamin K and thereby depletion of coagulation proteins.^18^ The anticoagulant effects of VKA are delayed until the already present coagulation factors are sufficiently depleted. Since factor VII has the shortest half-life (4–6 hours), coagulopathy can be expected 12–16 hours after ingestion of a VKA.^19^

In a retrospective study of acute warfarin overdoses in adults, it was found that a single, high dose (more than 10 mg) of vitamin K was not a guarantee to reverse or prevent worsening of coagulopathy.^3^ This worsening might be due to the ongoing absorption of excessive VKA (in case of bezoar) in combination with the short half-life of vitamin K (1.5–2.0 hours^20^). However, vitamin K seems to be available longer for the synthesis of new coagulation factors than can be expected based on this half-life. As shown in adult patients receiving vitamin K for the correction of supratherapeutic warfarin levels, a single dose of vitamin K resulted in a steady decline of the INR over 48 hours.^21^ This illustrates an uncertainty regarding the optimal dosage frequency in case of acute VKA intoxications.

Since we expected the absorption and the half-life of acenocoumarol (normally 8–14 hours) to be significantly prolonged due to the large number of ingested tablets, we decided to repeat the intravenous dose of 5 mg vitamin K after 12 hours. We decided to only administer extra vitamin K again, depending on a possible rise of the INR, which turned out to be not necessary. In acute intoxications with compounds with a longer half-life than acenocoumarol (eg, warfarin, with a half-life of 40 hours), the need for more administrations of vitamin K can be expected.

**Learning points**

- In this paediatric case, successful prevention of coagulopathy after the ingestion of a high dose of acenocoumarol was achieved by the administration of intravenous vitamin K and activated charcoal.
- In similar cases, we suggest administering two doses of intravenous vitamin K of 0.3 mg/kg per dose (maximum of 10 mg) with an interval of 12 hours. Additional administrations of vitamin K depend on the international normalised ratio (INR), measured at least every 12 hours in the first 48 hours.
- If elevated, the INR should be monitored for at least 48 hours after the last administration of vitamin K, to exclude coagulopathy.
